# Hydroxychloroquin – nicht immer ein harmloses Medikament für den Off-label-Gebrauch in der Dermatologie

**DOI:** 10.1007/s00105-024-05294-y

**Published:** 2024-01-30

**Authors:** Paloma Seidel, Eva Spukti, Kerstin Steinbrink, Dieter Metze, Markus Böhm

**Affiliations:** Klinik für Hautkrankheiten, Universitätsklinikum Münster, Von-Esmarch-Str. 58, 48149 Münster, Deutschland

**Keywords:** DRESS-Syndrom, Arzneimittelreaktion, COVID-19, Off-label-Medikation, Lichen planopilaris, DRESS syndrome, Drug-related side effects and adverse reactions, COVID-19, Off-label medication, Lichen planopilaris

## Abstract

Hydroxychloroquin wird bei Entzündungserkrankungen eingesetzt und gilt als nebenwirkungsarm. Wir berichten über eine Patientin, die sich mit einem schweren Exanthem nach Einnahme von Hydroxychloroquin vorstellte, das sie im Rahmen eines Lichen planopilaris erhielt. Basierend auf klinischen, laborchemischen und histologischen Befunden, wurde die Diagnose einer DRESS(„drug reaction with eosinophilia and systemic symptoms“)-artigen Arzneimittelreaktion gestellt. Der Fall illustriert, dass Hydroxychloroquin in seltenen Fällen zu schweren unerwünschten Wirkungen führen kann und Patienten, die dieses Medikament nehmen, sorgfältig aufgeklärt werden müssen.

## Abstract (en)

Hydroxychloroquine is used for treatment of inflammatory diseases. It is considered to have few adverse effects. We report on a woman who developed a severe skin rash after intake of hydroxychloroquine, which she received for treatment of her lichen planopilaris. Based on the clinical, laboratory and histological findings the diagnosis of a drug reaction with eosinophilia and systemic symptoms (DRESS)-like drug reaction was established. Our case illustrates that hydroxychloroquine can lead to severe adverse effects in rare cases and that patients receiving this drug must be thoroughly informed.

## Anamnese

Eine 51-jährige Patientin stellte sich im September 2020 mit einem neu aufgetretenen, progredienten, stark juckenden Exanthem am gesamten Integument vor. Des Weiteren gab die Patientin eine Schwellung der Lippen und ein leichtes Globusgefühl am Hals an. Aufgrund eines Lichen planopilaris sei vor 16 Tagen eine Therapie mit Hydroxychloroquin (200 mg 1–0–1) als Off-label-Medikation initiiert worden. Diese Medikation sei am Tag zuvor bei beginnendem Exanthem abgesetzt worden. Ansonsten bestünden bis auf eine Hypothyreose, die mit L‑Thyroxin behandelt wurde, keine weiteren Erkrankungen und keine weitere Medikation.

## Befund

**Figure 1:**
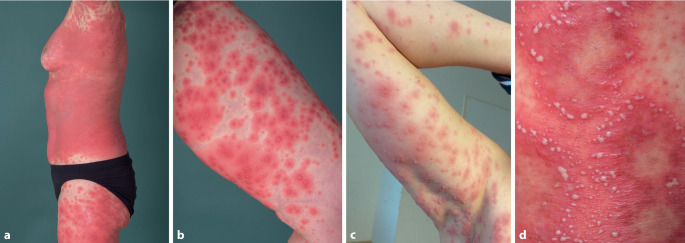

Es imponierte ein polymorphes Bild aus großflächig konfluierenden, teils düsterroten Erythemen, disseminierten Pusteln, Vesikeln und Blasen sowie teils kokardenförmigen Plaques am gesamten Integument unter Aussparung der Schleimhäute (Abb. 1a–d). Zudem zeigte sich eine dezente Schwellung im Bereich der Lippen.

## Diagnose

**Figure 2:**
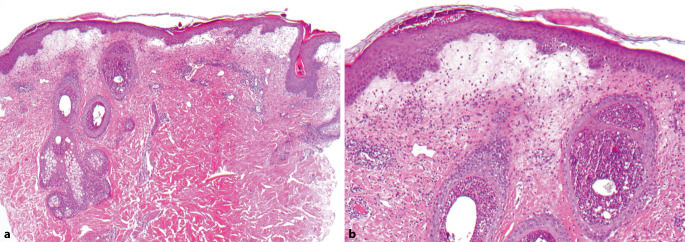

Es erfolgten eine Blutentnahme, Abstriche aus einer Vesikula bzw. Pustel sowie die Entnahme einer Hautbiopsie am rechten Schulterblatt. Laborchemisch zeigten sich eine Leukozytose (bis 19,93 Tsd/µl) sowie eine Erhöhung der Leberparameter (GOT [Glutamat-Oxalacetat-Transaminase] 57 U/l, GPT [Glutamat-Pyruvat-Transaminase] 84 U/l, gamma-GT [Gamma-Glutamyl-Transferase] 62 U/l) bei fehlender Eosinophilie. Eine umfangreiche Virusdiagnostik (HSV[Herpes-simplex-Virus]1, HSV2, VZV [Varizella-Zoster-Virus], Enteroviren, SARS-CoV-2) und die Syphilisdiagnostik waren negativ. Histologisch fanden sich in der Epidermis und in dilatierten Haarfollikelostien Ansammlungen von neutrophilen Granulozyten, subepidermal ein Ödem und perivaskulär akzentuiert lymphozytär betonte Entzündungszellinfiltrate mit Beimengung von neutrophilen Granulozyten. In der PAS(„periodic acid–Schiff reaction“)-Färbung waren keine Pilzelemente zu erkennen (Abb. 2a, b). Die direkte Immunfluoreszenz zeigte keine Antikörperablagerungen. Eine Sonographie von Abdomen und ein Röntgen des Thorax waren ohne pathologischen Befund. Die Patientin fieberte kurzzeitig auf (38,4 °C). In Zusammenschau von Anamnese, klinischen, laborchemischen und histologischen Befunden konnte die Diagnose einer DRESS(„drug reaction with eosinophilia and systemic symptoms“)-artigen Arzneimittelreaktion nach Einnahme von Hydroxychloroquin gestellt werden.

## Therapie und Verlauf

Es erfolgte eine hoch dosierte orale Steroidstoßtherapie mit Prednisolon, wobei die Dosis aufgrund des persistierenden starken Eruptionsdruckes mehrmals angepasst werden musste (bis zu 2 mg/kgKG [Körpergewicht]). Des Weiteren wurde topisch mit Clobetasol-17-propionat-Creme am Rumpf und an den Extremitäten, mit Methylprednisolonaceponat-Creme im Gesicht und Clobetasol-17-propionat-Lösung am Capillitium therapiert. Während des 4‑wöchigen Krankenhausaufenthaltes zeigten die Hautveränderungen einen undulierenden 2‑gipfligen Verlauf in kraniokaudaler Richtung. Nach Einleitung einer zusätzlichen Behandlung mit Dapson (50 mg 1‑mal täglich für 7 Tage, dann 2‑mal täglich) zeigte sich der Hautbefund langsam, aber vollständig regredient. In den folgenden Monaten zeigte sich bei den Verlaufskontrollen ein weiterhin stabiler Hautbefund, sodass die Dosis von Dapson sukzessiv reduziert und die Therapie im Juni 2021 abgesetzt werden konnte und die Medikation mit Prednisolon ausgeschlichen und im Mai 2021 beendet wurde. Hinweise auf Virusreaktivierungen, z. B. durch EBV (Epstein-Barr-Virus), oder eine kardiale Beteiligung ergaben sich im Verlauf nicht.

## Diskussion

Das DRESS(„drug reaction with eosinophilia and systemic symptoms“)-Syndrom ist eine schwere, T‑Zell-mediierte Arzneimittelreaktion, die typischerweise 2 bis 6 Wochen nach Beginn einer Systemtherapie beginnt und mit Fieber, einem generalisierten Exanthem, Lymphknotenschwellungen und hämatologischen Auffälligkeiten (insbesondere Leukozytose, Eosinophilie) einhergeht [4, 6]. Ätiopathogenetisch werden eine genetische Prädisposition und eine virale Reaktivierung (insbesondere HHV[humanes Herpesvirus]-6) diskutiert [8]. Die Klinik und insbesondere die Effloreszenzen sind heterogen, wobei typischerweise über 50 % der Körperoberfläche betroffen sind [8]. Eine Beteiligung von Leber, Niere und anderen Organen ist möglich, und die Erkrankung kann sogar letal verlaufen [3]. Umso wichtiger ist es daher, mögliche Auslöser zu identifizieren, um die Therapie mit dem kausalen Medikament zügig zu beenden und eine konsequente Systemtherapie mit Steroiden einzuleiten. Unser Fall zeigt, dass Hydroxychloroquin selten als Auslöser einer DRESS-artigen Arzneimittelreaktion fungieren kann. So sind bei unserer Patientin die RegiSCAR-Kriterien nach Hama et al. nicht erfüllt [5]. Es sind mindestens 20 weitere ähnliche, schwere kutane Reaktionen auf Hydroxychloroquin, die 2 bis 3 Wochen nach Therapiebeginn einsetzten, beschrieben [7]. Die Bezeichnung dieser Reaktionen ist in der Literatur uneinheitlich [7].

Hydroxychloroquin erlangte im Rahmen der COVID-19-Pandemie für die Allgemeinbevölkerung breitere mediale Aufmerksamkeit, da es als scheinbar harmloses und möglicherweise antivirales Mittel von Donald Trump mit den Worten „What do you have to lose? Take it!“ proklamiert wurde [1, 9].

Hydroxychloroquin wird aufgrund seiner immunmodulierenden und antiinflammatorischen Eigenschaften bei vielen Entzündungserkrankungen – teilweise als Off-label-Medikation – angewandt und gilt als Medikament mit günstigem Sicherheitsprofil [2]. Unser Fall macht jedoch deutlich, dass Hydroxychloroquin nicht leichtfertig als harmloses Medikament zu werten ist.

## Fazit für die Praxis

- Hydroxychloroquin kann in seltenen Fällen zu schweren Arzneimittelreaktionen der Haut führen.
- Eine vollständige Aufklärung von Patienten, die dieses Medikament gerade als Off-label-Medikation erhalten, ist immer nötig.
